# Lamb Meat Quality and Carcass Evaluation of Five Autochthonous Sheep Breeds: Towards Biodiversity Protection

**DOI:** 10.3390/ani11113222

**Published:** 2021-11-11

**Authors:** Maria Giovanna Ciliberti, Antonella Santillo, Rosaria Marino, Elena Ciani, Mariangela Caroprese, Luigina Rillo, Donato Matassino, Agostino Sevi, Marzia Albenzio

**Affiliations:** 1 Department of Agriculture, Food, Natural Resources and Engineering (DAFNE), University of Foggia, 71122 Foggia, Italy; maria.ciliberti@unifg.it (M.G.C.); antonella.santillo@unifg.it (A.S.); rosaria.marino@unifg.it (R.M.); mariangela.caroprese@unifg.it (M.C.); agostino.sevi@unifg.it (A.S.); 2 Department of General and Environmental Physiology, University of Bari, 70125 Bari, Italy; elena.ciani@uniba.it; 3 Consortium for Experimentation, Dissemination, and Application of Innovative Biotechniques, ConSDABI NFP, I FAO-GS AnGR, 82100 Benevento, Italy; luiginarillo1@gmail.com (L.R.); matassinod@consdabi.org (D.M.)

**Keywords:** lamb, autochthonous breeds, carcass evaluation, conjugated linoleic acid, biodiversity, sustainability

## Abstract

**Simple Summary:**

The biodiversity protection represents a challenge of the agenda ONU 2030 for Sustainable Development Goals (SDG). Autochthonous sheep breeds, including Altamurana, Bagnolese, Gentile di Puglia, Laticauda and Leccese, reared in continental Southern Italy, are strongly affected by extinction risk; thus, it is urgent to find new solutions to valorise their products and obtain sustainable and smart food from local farms. The objective of the present study was to assess the lamb carcass commercial quality, chemical and fatty acid composition of Altamurana, Bagnolese, Gentile di Puglia, Laticauda and Leccese autochtonous sheep breeds. Data on EU Mediterranean classification showed that the carcasses from both Bagnolese and Laticauda breeds were classified in the heaviest category C. Moreover, breed can influence the meat fatty acids profile, which is resulted rich in conjugated linoleic acid with good nutritional properties; thus, lambs’ meat from autochthonous sheep breeds can be considered interesting for human consumption. The present study can help to protect the biodiversity of livestock heritage of Southern Italy and open a new field for the valorisation and promotion of their derived products.

**Abstract:**

In the present study, the evaluation of the carcasses and meat quality, in terms of chemical composition and fatty acid profile, of lambs from five autochthonous sheep breeds (Altamurana, Bagnolese, Gentile di Puglia, Laticauda, and Leccese) reared in continental Southern Italy, were studied. All the carcasses were evaluated according to the EU Mediterranean classification system for carcasses weighing less than 13 kg. Meat chemical composition and fatty acids profile were assessed on both loin and leg commercial cuts. Fatty acid composition of loin resulted in differences among breeds, displaying lower values of saturated fatty acid in Altamurana, Bagnolese, and Leccese breeds and the highest content of polyunsaturated fatty acid in the Altamurana breed. Principal component analysis grouped lamb according to fatty acid content and to conjugated linoleic acid (CLA), omega n-3 and n-6 fatty acids; thus, Altamurana, Bagnolese, and Leccese breeds are characterized by the highest values of CLA content. Our data demonstrated that lamb meat from autochthonous breed has good carcass quality and the content of CLA, n-3, and n-6 was valuable for human consumption; therefore, the valorisation of local meat quality can help to avoid the extinction of the autochthonous breed offering to the market and consumer’s high nutritive products.

## 1. Introduction

Survival of a breed is linked to its ability to meet current and future market demand [1]. Biodiversity protection is considered of great importance, apart from as genetic reservoir of native breed, strictly connected to cultural land heritage, for future breeding options, but especially for preserving the environment, the landscape, the human presence in marginal areas [2].

Sheep are characterized by the greatest variety and large number of breeds mainly reared in Europe; where the sustainability issues need to be covered to obtain small ruminants’ production more resilient and competitive from an economic point of view [3]. In Southern Italy, autochthonous breeds reared for lamb production are usually slaughtered at a young age to meet the consumer market demand of light lambs. In Apulia and Campania regions of Continental Southern Italy, most represented breeds are Altamurana, Bagnolese, Gentile di Puglia, Laticauda and Leccese that are autochthonous breeds with a strict relationship with landscape and traditions. According to National Data Bank of the Zootechnical Registry (BDN), established by the Italian Ministry of Health, in the recent census (30 June 2021, Figure 1) the Altamurana breed counted 779 heads, mainly distributed in Apulia region (89.73%); and the Leccese breed was about 1317 heads reared in the 90.96% in farms located in Apulia region. Laticauda and Bagnolese breeds were mostly reared in Campania region with 5085 heads distributed in small herds for Laticauda, and more than 17,982 heads spread in medium sized herds for Bognolese. Gentile di Puglia counted a total 10,664 heads on the national soil with 5551 heads in Apulia region, and the remaining flocks were distributed in the neighbouring regions as a heritage of ancient pastoral system, consisting in migrations of sheep and sheep-keepers from lowlands to uplands, to guarantee a grass-based feeding during the whole year. 

Lamb meat is characterized by valuable nutritional features which are well documented by numerous studies on fatty acid profile and on its role on human health [4,5]. Indeed, lamb meat is the richest meat source of conjugated linoleic acid (CLA) isomers that was associated with immune-modulating, anticarcinogenic and antiatherogenic properties, prevention of diabetes, and reduction of body fat, as reported in several studies performed both in vitro and on animals [6,7]. Furthermore, Nudda et al. [8] suggest the use of lamb meat in child’s diet as a source of essential fatty acids and for its apparent lower allergenicity compared with meat from other species, such as beef meat that registered an incidence occurrence of allergy between 3.28% and 6.52% among children with atopic dermatitis [9]. Fatty acid composition is influenced by both genetic and environmental factors. The effect of breed on lamb meat quality is controversial [10,11,12], thus it is important to assess on different sheep breeds in relation to animal performance and meat quality. In a recent study by Bittante et al. [13], autochthonous Alpine breeds were demonstrated to have good performances for producing meat when a total mixed diet instead of traditional method at pasture for fattening weaned lambs was performed. Notably, consumers are positively affected by the information concerning the local production of lamb and the animal welfare condition; thus, demonstrates that if the provenance is appropriately communicated to the consumers, the market of local sheep breed could be sustained [14]. 

Accordingly, our hypothesis was that in the biodiversity strategy is included the valorisation of the intrinsic local meat quality attributes, which should help the promotion and the implementation of new products in the market, preserving the extinction of the autochthonous breed and the conserving endangered genetic resources. 

Therefore, the aim of this study was to evaluate the carcass commercial quality, the chemical, and fatty acid composition of lamb meat obtained from five autochthonous sheep breeds reared in continental Southern Italy: Altamurana, Bagnolese, Gentile di Puglia, Laticauda, and Leccese. The relationship between carcass quality, with regard on fatness degree, and fatty acids profile was also investigated.

## 2. Materials and Methods

### 2.1. Animal Management and Meat Sampling

The experiment involved 50 lambs, ten from each of the following breeds reared in continental Southern Italy: Altamurana, Bagnolese, Gentile di Puglia, Laticauda, and Leccese. 

All animals were born in autumn and were suckled throughout the experimental period by their dams, which grazed on natural pasture. Lambs from each genotype were slaughtered at 50 ± 5 days of age. Animal handling followed the recommendations of EU (Directive 2010/63/EU) concerning animal care. The lambs were slaughtered according to industrial routines used in Italy and to the EU rule n. 1099/2009; and each carcass was weighted and chilled at 1–3 °C. Twenty-four hours after slaughtering, all carcasses were classified using the EU Mediterranean classification system. Fatness score and carcass colour were estimated by two trained assessors via the EU photographic standards for carcasses weighing ≤ 13.0 kg [15]. For degree of fatness assessment, each level of the EU scale (1-low; 4-high) was divided in three sub-levels, in order to detect small differences in the degree of fatness, to a conversion scale ranging from 1 to 12: very scarce (1 = 1, 2 = 1); scarce (3 = 1+, 4 = 2−, 5 = 2); medium (6 = 2+, 7 = 3−, 8 = 3); important (9 = 3+, 10 = 4−, 11 = 4); and high (12 = 4+). Colour evaluation was performed giving a score equal to 1 for carcasses with pink meat colour and score = 2 to carcasses having red meat colour. The carcass colour was also measured by a Minolta CR300 colorimeter (illuminant C) on the external side of muscles rectus abdominis, after skin removed; the following colour coordinates were measured: lightness (L*), redness (a*) and yellowness (b*). For each lamb carcass, three measurements were performed at different locations on the surface of the muscle. 

After carcass classification, loin and leg cuts were collected from one half of each carcass, vacuum packaged and stored at −20 °C until analysis. 

### 2.2. Animal Management and Meat Sampling

A food processor was used to mince meat in a homogeneous consistency. Proximate analysis in terms of moisture, protein, lipid, and ash content was performed in duplicate following the standard AOAC methods [16]. 

Extraction lipids methodology was in according to Bligh and Dyer [17]. Briefly, chloroform: methanol (15 mL, 1:2, *v*/*v*) solution was added to the sample (5 g) and homogenized for 2 min at 13,500 rpm using Ultra Turrax homogenizer (IKA T18 basic). Then, the homogenized sample was centrifuged (10 min, 1500 rpm) and the chloroformic phase was subjected to filtration step using anhydrous sodium sulphate. The collected sample was purified from chloroform using a rotary evaporator (Büchi Rotavapor R200/B490, Flawil, Switzerland) at 37 °C under vacuum condition. Subsequently, duplicate 100 mg of lipid extracted were methylated according to I.U.P.A.C. [18]. Gas-chromatograph analysis was performed using Agilent 6890N instrument coupled with a HP-88 fused-silica capillary column (length 100 m, internal diameter 0.25 mm, film thickness 0.25 μm). The following operating conditions were defined: a helium flow rate of 0.7 mL/min; a FID detector at 260 °C; a split-splitless injector at 220 °C with an injection rate of 120 mL/min and an injection volume of 1:1. The column temperature program was set for 4 min at 140 °C and then an increase to 220 °C at 4 °C/min. Both the retention time and area of each peak were calculated using the 6890N NETWORK GC system software. The retention time of the standards (FIM-FAME-37-Mix, Matreya, Pleasant Gap, PA, USA) was utilized for the identification of individual fatty acids methyl ester (FAME) peaks. 

Ulbricht and Southgate [19] formulas for the Atherogenic and Thrombogenic indices calculation were applied. 

### 2.3. Statistical Analysis

All the variables were tested for normal distribution using the Shapiro-Wilk test [20] and processed using ANOVA for repeated measures of SAS [21]. 

The model was (Equation (1):

yij= μ + αi+ βj + εij(1)

where yij is the observation ij; μ is the overall mean; α is the effect of genotype (i = 1–5); β is the effect of muscle (j = 1–2) and ε is the error. When significant effects were found (at *p* < 0.05), the Student t-test was used to locate significant differences between means. Linear simple correlations between fatness degree, intramuscular fat content and fatty acids percentage were performed. Principal component analysis (PCA) was performed by PRINCOMP procedure of SAS to obtain a visual representation of FAME and CLA isomer distribution in lamb meat from the five breeds.

## 3. Results and Discussion

### 3.1. Carcass Quality

Carcass characteristics of lambs from Altamurana, Bagnolese, Gentile di Puglia, Laticauda, and Leccese breed, are reported in Table 1. 

All carcasses fitted the Mediterranean scheme for carcasses weighing less than 13 kg. The Mediterranean classification system gives information on the conformation, fatness, and colour for the evaluation of lamb carcasses useful to fix meat market value.

Carcass weight differed among the five breeds; in particular, Bagnolese and Laticauda showed higher carcass weight than Altamurana, Gentile di Puglia, and Leccese breeds. Very light carcasses ascribed to category A (<7.0 kg) were found only in Altamurana and Leccese breeds represented by 20% and 10%. Moreover, 60% of Altamurana, 70% of Gentile di Puglia and 90% of Leccese carcasses were assigned to B category; whereas, all Bagnolese and 90% of Laticauda carcasses were classified in the heaviest category C. 

A breed effect was also found for degree of fatness showing the carcasses of heavier lambs the higher values of fatness degree. In the present survey, about 50% of lamb carcasses were placed in the highest weight category; thus, evidencing a good potential, for these autochthonous breeds, to produce heavier lambs with economic return for the stockbreeders.

Muscle from and Altamurana, Gentile and Leccese turned out to be paler and lighter as confirmed by lower colour score and higher L value than muscle from Laticauda. Furthermore, muscle from Bagnolese and Laticauda had higher value of redness compared to the other breed showing a darker meat. In the present survey, muscle colour parameters were affected by slaughtering weight according to Juárez et al. [22], who found that light lambs had a higher darker than suckling lambs. Values of colorimetric parameters are within the range of light pink meat belonging to first quality carcass [23]. 

### 3.2. Meat Quality 

Chemical composition of *Longissimus lumborum* (LL) samples from the five sheep breeds reared in continental Southern Italy are reported in Table 2. Protein content of lamb meat ranged between 21.55 ± 0.16% and 22.20 ± 0.16% and intramuscular lipid content ranged between 0.76 ± 0.11% and 1.77 ± 0.11% displaying differences among breeds. Total fat content indicates that meat from each breed may be classified as lean meat according to Food Advisory Committee [24] criteria (i.e., less than 5%). On average, in all breeds no significant differences were found in chemical composition (21.60 ± 0.07% and 21.36 ± 0.07% protein content in LL and leg, respectively, and 1.18 ± 0.05% and 1.09 ± 0.05% fat content in LL and leg, respectively, *p* > 0.05), and fatty acids profile (Table S1) between LL and leg cuts. Probably the lack of differences in the fatty acid profile of the sampled muscles could be ascribed to the poor depot of intramuscular fat due to the young age at slaughter of the lambs. No correlations between fatness degree, intramuscular fat content and fatty acids percentage were found. 

The proportion of fatty acid in the diet is very important in relation to consumer health, and its imbalance may increase the risk of coronary heart disease [4,25]. Fatty acid composition of LL samples, as the main representative muscle for meat chemical characterization, from the five sheep breeds Italy are reported in Table 3. The saturated fatty acid (SFA) percentage ranged from 38.8 to 43.5 ± 0.82 displaying the lowest values in Altamurana, Bagnolese, and Leccese lambs and the highest values in Gentile di Puglia and Laticauda lambs. Within SFA, several fatty acids evidenced different level among breeds. Meat from Leccese showed the highest value of caprylic (C8:0, *p* < 0.05) and capric acids (C10:0, *p* < 0.05); Gentile di Puglia showed the lowest percentage of miristic acid (C14:0, *p* < 0.01) and the highest value of stearic acid (C18:0, *p* < 0.001); whereas palmitic acid (C16:0, *p* < 0.001) was the lowest in Altamurana and Bagnolese breeds. It is well known that low level of lauric, miristic and palmitic fatty acids is linked to good dietary quality of meat; whereas, C12:0 to C18:0 fatty acids are correlated with the increase of serum triglyceride and cholesterol levels [26]. Notably, C18:0 was considered biologically neutral with respect to regulation of the concentration of cholesterol carried in low density lipoprotein [27,28]; thus, it was demonstrated that stearic acid cannot directly implies the increase of the risk for cardiovascular diseases. 

Breed significantly influenced also unsaturated fatty acids profile of lamb meat. Monounsaturated fatty acids (MUFA) ranged from 35.94 to 41.18 ± 0.75%, and polyunsaturated fatty acids (PUFA) ranged from 17.17 to 24.72 ± 0.49%, with the lowest and the highest percentage in Altamurana lamb meat, respectively. Trans vaccenic acid (C18:1 trans-11) was higher in Altamurana, Bagnolese, and Leccese meat compared to other breeds; while oleic acid (C18:1 cis-9) showed the highest level in Laticauda meat. Within PUFA, meat from Altamurana lambs showed the highest content of alpha-linolenic acids (C18:3 n3) and eicosapentaenoic acid (EPA, C20:5 n3); moreover, Altamurana, Bagnolese and Leccese showed the highest docoesaenoic acid (DHA, C22:6 n3) and the lowest linoleic acids (C18:2 n6) percentage. MUFA and PUFA are considered protective factors with the former being able to reduce low density lipoprotein concentration in blood and the latter being involved in the reduction of risk of human coronary heart disease [4]. Meat from Altamurana, Bagnolese and Leccese lambs displayed the highest CLA percentage. The ability to produce CLA in milk is related to two broad aspects: the rumen output of C18:1 trans-11 and, to a lesser extent, of C18:2 cis-9 trans-11, and the amount and activity Δ^9^-desaturase in tissues [29]. However, endogenous synthesis also occurs in the subcutaneous or intramuscular fat by the action of Δ^9^-desaturase [30]. It is well known that CLA isomers are found in food from ruminant as intermediate products of the biohydrogenation of linoleic acid in the rumen.

The real contribution of breed in meat fatty acid composition is difficult to assess. Indeed, the comparison between breeds can be confounded by fat level, live weight or age at slaughter and production system effects [31,32]. In the present study, the differences emerged in fatty acid composition between breeds could be related to the fat content; indeed, in Gentile di Puglia and Laticauda meat, a concomitant high level of both SFA and MUFA and intramuscular fat content was observed. Accordingly, De Smet et al. [30] reported that the content of SFA and MUFA increase faster with increasing fatness than PUFA content. 

Irrespective to the breeds, meat of the five investigated sheep breeds showed higher content of PUFA than values reported by Diaz et al. [33] in Rasa Aragonese lambs (15.58%) and by Oriani et al. [34] in Italian Merino lambs (from 12.87 to 16.09%). In ruminants, PUFA deposit typically occurred in phospholipids [35]; therefore, very lean breeds could develop relatively high amounts of PUFA, as compared with fatter lambs, in which the phospholipid proportion can be diluted by an increasing level of neutral storage lipid.

Conjugated linoleic acid and nutritional indexes of the intramuscular lipid fraction of the five breeds were reported in Table 4. 

Polyunsaturated to saturated fatty acids ratio (P/S) is usually used as an index of the nutritional value of dietary fat with the recommended level in human diet of 0.45; values lower than this threshold are considered unfavourable because they may induce an increase in cholesterolaemia [36]. For many years, nutritionists have focused on the type of PUFA in meat, and the balance in the diet between n-6 and n-3 PUFA is involved in the prevention of cancer and heart diseases [25,35]. 

Meat from Altamurana, Bagnolese and Leccese lambs displayed a n-6/n-3 ratio lower than the recommended threshold of 4.0 [36]. AI and TI indexes express the risk associated to develop atherosclerosis and thrombosis upon feed intake; therefore, when their levels are lower, the nutritional features and health properties of food are considered better. Irrespective of breed effect, the values reported in lamb meat for both indexes were lower than those reported by other authors in lambs slaughtered at similar age [12,34,37]. When comparing breeds, Altamurana, Bagnolese and Gentile di Puglia displayed the lowest AI and Altamurana and Bagnolese meat the lowest TI. 

**Figure 2:**
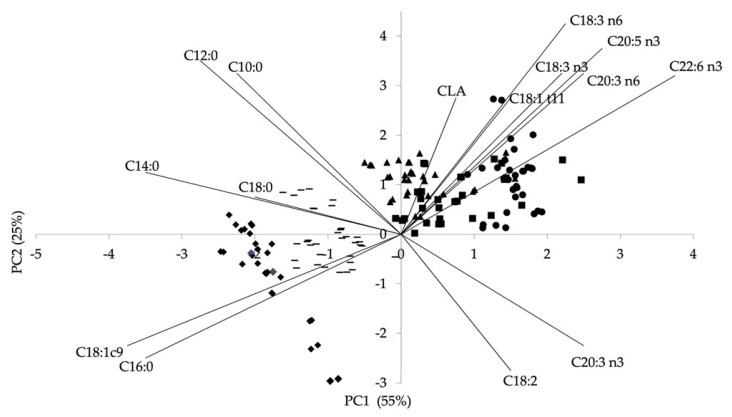
Principal component analysis of the fatty acid methyl ester (FAME) and conjugated linoleic acid (CLA) in *Longissimus lumborum* (LL) muscle of lambs from five breeds reared in continental Southern Italy. ● Altamurana; ■ Bagnolese; ▲ Leccese; ◆ Gentile di Puglia; ▬ Laticauda.

Principal component analysis of fatty acids in lamb meat of the five sheep breeds is reported in Figure 2. 

The PCA analysis performed to FAME and CLA was useful to distinguish lamb meat based on its nutritional properties. Saturated fatty acid content was the main factor explained by the first principal component, whereas the content of CLA and PUFA n-3 and n-6 fatty acids were the dominating factors along the second principal component. Meat samples from Gentile di Puglia and Laticauda breeds were located in a defined zone of the plot distinguished from one another along the first principal component; Leccese, Altamurana and Bagnolese clustered in the same area characterized by a positive loading both on the first and on the second component owing to the greater weight of CLA and n-3 and n-6 fatty acids on the lamb meat lipid profile. 

## 4. Conclusions

The present study aimed at preserving the extinction and conserving endangered genetic resources thorough the characterization and valorisation of meat nutritional properties from five autochthonous sheep breeds of Southern Italy. All the breeds involved in this study showed valuable nutritional features of the meat, particularly the fatty acid profile resulted very interesting for human health, although differences among breeds appeared. Moreover, according to EU Mediterranean classification system, about 50% of lamb carcasses exhibited a good carcass fatness score; thus, demonstrated the potentiality of autochthonous breed to produce heavier lambs with an important economic return for farmers. Future direction will address the discovering of innovative products from sheep autochthonous breed covering the biodiversity pillars and the more sustainable food production passing through from the tradition to the innovation.

## Figures and Tables

**Figure 1:**
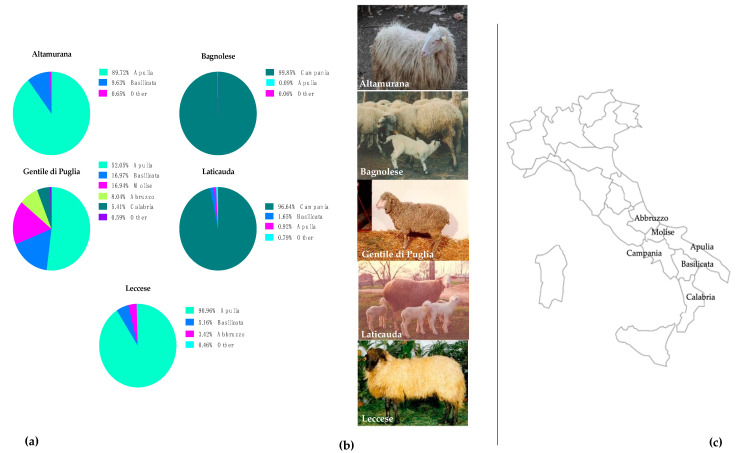
(**a**) Parts of whole representation in percentage of the five sheep breed most represented in Southern Italy; Altamurana, Bagnolese, Gentile di Puglia, Laticauda and Leccese according to the last census (30 June 2021) from the National Data Bank of the Zootechnical Registry (BDN); (**b**) pictures of Altamurana, Bagnolese, Gentile di Puglia, Laticauda and Leccese by https://www.agraria.org/ovini (accessed on 15 October 2021) (**c**) Southern Italy regions’ maps mainly representative of the autochthonous sheep breeds.

**Table 1:** Carcass traits of breeds reared in continental Southern Italy according to the EU classification system for light lambs (means ± SEM).

				Breed				
Item		Altamurana	Bagnolese	Gentile di Puglia	Laticauda	Leccese	SEM	Effect, *p*
Carcass weight, kg		9.51 a	11.78 b	9.13 a	11.96 b	8.13 a	0.46	**
Category ^1^ (n cacasses)	A	2				1		
	B	6		7	1	9		
	C	2	10	3	9			
Fatness degree, score		3.00 ab	3.67 b	3.20 b	4.56 c	2.21 a	0.26	**
Colour, score ^2^		1.10 a	1.60 b	1.20 ab	1.50 b	1.10 a	0.13	*
L*		46.08 b	45.61 ab	46.23 b	44.83 a	47.12 b	0.35	*
a*		16.76 a	17.25 ab	16.58 a	17.37 b	16.45 a	0.27	*
b*		9.35	8.75	9.11	8.81	9.55	0.25	NS

NS: not significant; *: *p* < 0.05; **: *p* < 0.01. a, b means with different letters are significantly different (*p* < 0.05). ^1^ Carcass category distribution (n = 10 animals for each breed) according to Mediterranean classification, where A = final body weight < 7.0 kg, B = final body weight ranged from 7.1 to 10 kg, C = final body weight ranged from 10.1 to 13 kg. ^2^ Colour score: lightness (L*), redness (a*) and yellowness (b*).

**Table 2:** Chemical composition (%) of *Longissimus lumborum* (LL) muscle of lambs from five sheep breeds reared in continental Southern Italy (means ± SEM).

	Breed						
Item	Altamurana	Bagnolese	Gentile di Puglia	Laticauda	Leccese	SEM	Effect, *p*
Moisture	76.08	75.76	75.49	74.72	75.96	0.51	NS
Protein	21.95 ab	22.05 b	21.85 ab	22.20 b	21.55 a	0.16	**
Fat	0.76 a	0.94 ab	1.44 cd	1.77 d	1.14 bc	0.11	***
Ash	1.21	1.25	1.22	1.31	1.35	0.08	NS

NS: not significant; **: *p* < 0.01; ***: *p* < 0.001. a, b, c, d means with different letters are significantly different (*p* < 0.05).

**Table 3:** Intramuscular fatty acids content (g/100 g fatty acid methyl esters) of *Longissimus lumborum* (LL) muscle of lambs from five sheep breeds reared in continental Southern Italy (means ± SEM).

			Breed				
Item	Altamurana	Bagnolese	Gentile di Puglia	Laticauda	Leccese	SEM	Effect, *p*
C8:0	0.01 a	0.03 a	0.01 a	0.01 a	0.15 b	0.02	*
C10:0	0.24 a	0.26 a	0.14 a	0.26 a	0.51 b	0.04	*
C12:0	0.59	0.45	0.41	0.51	0.62	0.08	NS
C14:0	4.21 b	4.31 b	3.16 a	4.63 b	5.21 b	0.33	**
C14:1	0.38	0.34	0.21	0.34	0.38	0.11	NS
C15:0	0.59	0.41	0.39	0.49	0.47	0.08	NS
C16:0	18.55 a	18.82 a	21.08 b	23.29 c	20.48 c	0.49	***
C16:1	0.96 a	1.48 b	1.27 b	1.75 c	1.64 c	0.08	***
C17:0	0.59 a	0.78 b	0.68 b	0.99 b	0.41 a	0.06	***
C17:1	0.51 b	0.47 b	0.36 a	0.64 c	0.59 b	0.1	NS
C18:0	14.82 b	12.47 a	16.64 c	12.78 a	12.52 a	0.27	***
C18:1 11t	6.67 b	5.93 b	4.65 a	3.88	5.96 b	0.41	***
C18:1 9c	26.6 a	30.36 b	30.72 b	32.67 c	31.78 b	0.64	***
C18:2 n6	8.28 a	8.41 a	11.05 b	10.08 b	6.93 a	0.51	***
C18:3 n-6	0.57 c	0.26 b	0.15 a	0.26 b	0.41 c	0.03	***
C18:3 n-3	3.76 c	3.41 c	1.29 a	1.57 a	2.67 b	0.14	***
C21:0	0.22	0.33	0.11	0.10	0.11	0.07	NS
C20:3 n-6	0.42 c	0.26 b	0.15 a	0.26 b	0.41 c	0.05	**
C20:4 n-6	3.83 c	3.25 b	2.98 b	3.24 b	2.38 a	0.14	*
C20:5 n-3	2.89 c	2.04 b	0.47 a	0.33 a	1.71 b	0.15	***
C22:6 n-3	1.33 b	1.18 b	0.44 a	0.34 a	1.11 b	0.08	***
CLA tot	3.18 b	3.07 b	1.51 a	1.21 a	2.96 b	0.08	***
SFA	40.05 a	38.90 a	42.61 b	43.47 b	40.81 a	0.82	***
MUFA	35.45 a	38.89 b	38.99 b	39.36 b	40.85 b	0.75	*
PUFA	24.65 c	21.91 b	18.40 a	17.17 a	18.37 a	0.49	**

NS: not significant; *: *p* < 0.05; **: *p* < 0.01; ***: *p* < 0.001. a, b, c means with different letters are significantly different (*p* < 0.05). C8:0 = Caprylic acid; C10:0 = Capric acid; C12:0 = Lauric acid; C14:0 = Myristic acid; C14:1 = Myristoleic acid; C15:0 = Pentadecylic acid; C16:0 =Palmitic acid; C16:1 = Palmitoleic acid; C17:0 = Heptadecanoic acid; C17:1 = Heptadecenoic acid; C18:0 = Stearic acid; C18:1 11t = trans-Vaccenic acid; C18:1 9c = Oleic acid; C18:2 n6 = Linoleic acid; C18:3 n-6 = gamma-Linolenic Acid; C18:3 n-3 = alpha-Linolenic Acid; C21:0 = Heneicosanoic acid; C20:3 n-6 = Dihomo-gamma-linolenic acid; C20:4 n-6 = Arachidonic acid; C20:5 n-3 = Eicosapentaenoic Acid; C22:6 n-3 = Docosahexaenoic Acid; CLA tot = Sum of Conjugated linoleic acid (CLA) 9c11t, CLA 10t12c, and CLA 9t11t; SFA = Saturated fatty acids, MUFA = Monounsaturated fatty acids, PUFA = Polyunsaturated fatty acids.

**Table 4:** Intramuscular conjugated linoleic acid CLA content (g/100 g fatty acid methyl esters) and nutritional indices of *Longissimus lumborum* (LL) of lambs from five sheep breeds reared in continental Southern Italy (means ± SEM).

			Breed				
Item	Altamurana	Bagnolese	Gentile di Puglia	Laticauda	Leccese	SEM	Effect, *p*
P/S	0.61 b	0.56 b	0.43 a	0.39 a	0.45 a	0.03	**
n-6	16.28 c	15.25 b	15.85 bc	15.05 b	13.09 a	0.32	***
n-3	7.98 d	6.62 c	2.21 a	2.24 a	5.48 b	0.16	***
n-6/n-3	2.04 a	2.30 a	7.20 c	6.72 b	2.38 a	0.13	**
AI ^1^	0.61 a	0.60 a	0.62 a	0.75 b	0.71 ab	0.04	*
TI ^2^	0.75 a	0.75 a	1.23 c	1.20 c	0.88 b	0.04	***

*: *p* < 0.05; **: *p* < 0.01; ***: *p* < 0.001. ^1^ AI = Atherogenic Index: (C12:0 + 4 × C14:0 + C16:0)/[(ΣMUFA + ΣPUFA (ω-6) and (ω-3)]; ^2^ TI = Thrombogenic Index: (C14:0 + C16:0 + C18:0)/[(0.5 × ΣMUFA + 0.5 × ΣPUFA (ω-6) + 3 × ΣPUFA (ω-3) + (ω-3)/(ω-6)]. a, b, c means with different letters are significantly different (*p* < 0.05). P/S = polyunsaturated fatty acids/saturated fatty acids ratio, n-3 = omega-3 fatty acids, n-6 = omega 6 fatty acids, n-6/n-3 = omega-6 fatty acids/omega 3 fatty acids ratio.

## Data Availability

Data supporting the findings of this study are available from the corresponding author upon reasonable request.

## Supplementary Materials

The following are available online at https://www.mdpi.com/article/10.3390/ani11113222/s1, Table S1: Average of the intramuscular fatty acids content (g/100 g fatty acid methyl esters) of *Longissimus lumborum* (LL) and Leg muscles of lambs (means ± SE).
