# Supplementary material for: Lamb Meat Quality and Carcass Evaluation of Five Autochthonous Sheep Breeds: Towards Biodiversity Protection
Source: Animals (Basel). 2021 Nov 11;11(11):3222. doi: 10.3390/ani11113222 (PMC8614373; doi:10.3390/ani11113222)
Supplement: Supplementary file 1 [file animals-11-03222-s001.zip › animals-1405707-supplementary.pdf]

**Supplementary Table S1.** Average of the intramuscular fatty acids content (g/100 g fatty acid methyl esters) of *Longissimus lumborum* (LL) and Leg muscles of lambs (means ± SE).

| Item | LL | Leg | Effect, *p* |
|---|---|---|---|
| C8:0 | 0.034 ± 0.007 | 0.027 ± 0.004 | 0.37 |
| C10:0 | 0.283 ± 0.02 | 0.249 ± 0.009 | 0.19 |
| C12:0 | 0.492 ± 0.02 | 0.482 ± 0.02 | 0.73 |
| C14:0 | 4.308 ± 0.16 | 4.272 ± 0.13 | 0.87 |
| C14:1 | 0.327 ± 0.01 | 0.339 ± 0.01 | 0.47 |
| C15:0 | 0.469 ± 0.02 | 0.339 ± 0.01 | 0.15 |
| C16:0 | 20.486 ± 0.28 | 19.876 ± 0.27 | 0.12 |
| C16:1 | 1.369 ± 0.04 | 1.427 ± 0.04 | 0.36 |
| C17:0 | 0.693 ± 0.03 | 0.744 ± 0.02 | 0.20 |
| C17:1 | 0.533 ± 0.02 | 0.553 ± 0.02 | 0.46 |
| C18:0 | 13.829 ± 0.21 | 13.606 ± 0.16 | 0.39 |
| C18:1 11t | 5.016 ± 0.24 | 5.467 ± 0.19 | 0.15 |
| C18:1 9c | 30.508 ± 0.41 | 30.562 ± 0.37 | 0.93 |
| C18:2 n6 | 8.847 ± 0.231 | 9.159 ± 0.408 | 0.65 |
| C18:3 n-6 | 0.325 ± 0.12 | 0.353 ± 0.02 | 0.31 |
| C18:3 n-3 | 2.107 ± 0.16 | 2.315 ± 0.17 | 0.37 |
| C21:0 | 0.155 ± 0.02 | 0.194 ± 0.02 | 0.20 |
| C20:3 n-6 | 0.336 ± 0.02 | 0.305 ± 0.01 | 0.26 |
| C20:4 n-6 | 3.939 ± 0.27 | 3.765 ± 0.13 | 0.56 |
| C20:5 n-3 | 1.459 ± 0.12 | 1.510 ± 0.12 | 0.77 |
| C22:6 n-3 | 0.869 ± 0.05 | 0.975 ± 0.07 | 0.21 |
| CLA tot | 2.360 ± 0.13 | 2.389 ± 0.11 | 0.87 |
| SFA | 44.389 ± 0.39 | 40.689 ± 0.34 | 0.18 |
| MUFA | 37.845 ± 0.52 | 38.416 ± 0.33 | 0.36 |
| PUFA | 20.769 ± 0.73 | 20.828 ± 0.56 | 0.95 |

$p>0.05$ not significant, C8:0 = Caprylic acid; C10:0 = Capric acid; C12:0 = Lauric acid; C14:0 = Myristic acid; C14:1 = Myristoleic acid; C15:0 = Pentadecylic acid; C16:0 =Palmitic acid; C16:1 = Palmitoleic acid; C17:0 = Heptadecanoic acid; C17:1 = Heptadecenoic acid; C18:0 = Stearic acid; C18:1 11t = trans-Vaccenic acid; C18:1 9c = Oleic acid; C18:2 n6 = Linoleic acid; C18:3 n-6 = gamma-Linolenic Acid; C18:3 n-3 = alpha-Linolenic Acid; C21:0 = Heneicosanoic acid; C20:3 n-6 = Di-homo-gamma-linolenic acid; C20:4 n-6 = Arachidonic acid; C20:5 n-3 = Eicosapentaenoic Acid; C22:6 n-3 = Docosahexaenoic Acid; CLA tot= Sum of Conjugated linoleic acid (CLA) 9c11t, CLA 10t12c, and CLA 9t11t; SFA= Saturated fatty acids, MUFA= Monounsaturated fatty acids, PUFA = Polyunsaturated fatty acids.
